# Mn^2+^‐Activated Alkali Lithooxidosilicate Phosphors as Sustainable Alternative White‐Light Emitters

**DOI:** 10.1002/anie.202504078

**Published:** 2025-05-02

**Authors:** Lukas Maximilian Träger, Judith Ifeoma Ekeya, Annika Liesenfeld, Marc Wieczorek, Hubert Huppertz, Markus Suta

**Affiliations:** ^1^ Inorganic Photoactive Materials, Institute of Inorganic Chemistry Heinrich Heine University Düsseldorf 40225 Düsseldorf Germany; ^2^ Institute of General, Inorganic, and Theoretical Chemistry University of Innsbruck Innrain 80–82 Innsbruck 6020 Austria

**Keywords:** Lithooxidosilicates, Lithosilicates, Manganese, Phosphors, Photoluminescence

## Abstract

Eu^2+^‐activated alkali lithooxidosilicates have emerged as promising candidates for narrow‐band cyan and green‐emitting phosphors for human‐centered lighting. Despite the vivid research on Eu^2+^‐activated alkali lithooxidosilicates, little to nothing is known about the possible luminescence with other activator ions than Eu^2+^. The more abundant transition metal ion Mn^2+^ is a potential alternative emitter with tunable emission over the visible spectral range. Mn^2+^‐activated alkali lithooxidosilicates show two emission bands in the green and red spectral range, respectively, making them intriguing candidates for one‐component phosphor‐converted white light‐emitting diodes. A total of seven Mn^2+^ activated compounds were prepared to elucidate possible trends. The results show that the ratio between green and red emission correlates to the size of the available activator sites and is easily controlled by the composition of the alkali lithooxidosilicate host compounds. Temperature‐dependent luminescence studies reveal that thermal quenching occurs slightly above room temperature for those compounds, which may be connected to the comparably low band gaps for silicates (*E*_g_ < 6 eV) and a consequent thermal ionization of excited electrons into the conduction band. Overall, this study comprises the first class of compounds with efficient tailored white‐light emission based on Mn^2+^ within one host compound.

## Graphical abstract

Eu^2+^‐activated alkali lithooxidosilicates are celebrated phosphor materials. We showed for the first time that the alternative, more abundant transition metal ion Mn^2+^ can also be incorporated into those compounds. The phosphors show two emission bands (green and red), making them interesting candidates for pc‐wLEDs with only one converting phosphor. The ratio of the two bands can be tailored by the composition of the host compound.

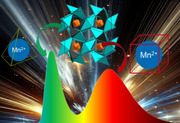

## Introduction

The quest for new, sustainable light sources is considered one major task to reduce human‐centered CO_2_ emissions. In recent years, phosphor‐converted light‐emitting diodes (pc‐LEDs) have emerged as promising candidates in this field as they combine low energy consumption and wide color tunability. Pc‐LEDs are usually constructed from an efficient blue‐emitting In_1‐_ *_x_* Ga*_x_* N chip, coated with one or more layers of phosphor materials, that convert the blue excitation radiation to light of longer wavelength.^[^ ^1^, ^2^, ^3^, ^4^, ^5^ ^]^ Today, an overwhelming number of phosphors for application in pc‐LEDs were introduced, including famous commercialized materials like Y_3_Al_5_O_12_:Ce^3+^ (YAG:Ce^3+/^ *λ*_em_ = 532 nm),^[^ ^6^, ^7^ ^]^ M_2_Si_5_N_8_:Eu^2+^ (M = Ca, Sr, Ba/*λ*_em_ = 580–610 nm),^[^ ^8^, ^9^, ^10^ ^]^ CaAlSiN_3_:Eu^2+^ (*λ*_em_ = 660 nm),^[^ ^11^ ^]^ K_2_SiF_6_:Mn^4+^ (*λ*_em_ = 620 nm),^[^ ^12^ ^]^ and *β*‐SiAlON:Eu^2+^ (*λ*_em_ = 535 nm).^[^ ^13^ ^]^ A particularly prominent emerging class of efficient phosphors are Eu^2+^‐activated UCr_4_C_4_‐type oxido‐ or nitridosilicates/‐aluminates, which have recently attracted much attention due to their (ultra‐)narrow emission bands with full widths at half maximum (FWHMs) below 2500 cm^−1^ at room temperature and amazing color‐tunability over the whole visible spectral range. Prominent representatives of this material class are Sr[LiAl_3_N_4_]:Eu^2+^ (SLA:Eu^2+^/*λ*_em_ = 654 nm/FWHM = 1180 cm^−1^),^[^ ^14^ ^]^ M[Mg_2_Al_2_N_4_]:Eu^2+^ (M = Ca, Sr, Ba/*λ*_em_ = 607–666 nm/FWHM = 1820–2330 cm^−1^),^[^ ^15^ ^]^ Sr[Mg_3_SiN_4_]:Eu^2+^ (*λ*_em_ = 615 nm/FWHM = 1170 cm^−1^),^[^ ^16^, ^17^ ^]^ Sr[Li_2_Al_2_O_2_N_2_]:Eu^2+^ (SALON:Eu^2+^/*λ*_em_ = 614 nm/FWHM = 1290 cm^−1^)^[^ ^18^ ^]^ and blue to green emitting alkali lithooxidosilicates like Na[Li_3_SiO_4_]:Eu^2+^ (*λ*_em_ = 469 nm/FWHM = 1460 cm^−1^),^[^ ^19^ ^]^ RbLi[Li_3_SiO_4_]_4_:Eu^2+^ (*λ*_em_ = 530 nm/FWHM = 1500 cm^−1^),^[^ ^20^, ^21^ ^]^ RbKLi_2_[Li_3_SiO_4_]:Eu^2+^ (*λ*_em_ = 474 nm, 532 nm/FWHM = 1100 cm^−1^, 1560 cm^−1^),^[^ ^22^ ^]^ and K_1.6_Na_2.1_Li_0.3_[Li_3_SiO_4_]_4_:Eu^2+^ (*λ*_em_  = 486 nm/FWHM = 890 cm^−1^).^[^ ^23^, ^24^ ^]^ Most recently, the solid solution Sr[Li_3_(Al_1‐_ *_x_* Ga*_x_*)O_4_] was discovered, being the first oxolithoaluminates and ‐gallates with UCr_4_C_4_‐related structure (*λ*_em_ = 554 nm/FWHM = 1589 cm^−1^ (*x* = 0), *λ*_em_  = 572 nm/FWHM = 1446 cm^−1^ (*x* = 1)).^[^ ^25^, ^26^ ^]^ Their formidable luminescence characteristics are closely related to the structural rigidity of the condensed anionic backbone that is made up from vertex‐ and edge‐sharing [MX_4_]‐tetrahedra (M = Li, Mg, Al, Si; X = O, N) forming *vierer*‐ring cation channels. The larger alkali or alkaline earth cations are located within these channels, occupying cuboidally coordinated sites.

Despite the success of Eu^2+^‐activated UCr_4_C_4_‐typed phosphors, reports on the introduction of other activator ions into UCr_4_C_4_‐typed materials are scarce and solely focused on trivalent lanthanides (typically Ce^3+^).^[^ ^16^, ^27^, ^28^, ^29^, ^30^, ^31^ ^]^ In view of more sustainable and abundant emitters, the 3d^5^ high spin (HS) configurated transition metal ion Mn^2+^ is a competitive alternative. The less extended nature of the 3d orbitals of Mn^2+^ compared to the 5d orbitals of Eu^2+^ could formally result in even narrower emission bands. Upon tetrahedral coordination by O^2−^ ligands, it can show thermally inert, narrow green emission, often with quenching temperatures above 600 K.^[^ ^32^ ^]^ Examples include MgAl_2_O_4_:Mn^2+^ (*λ*_em_ = 525 nm/FWHM = 1270 cm^−1^),^[^ ^33^ ^]^ ZnAl_2_O_4_:Mn^2+^ (*λ*_em_ = 510 nm/FWHM = 820 cm^−1^),^[^ ^32^, ^34^ ^]^ Zn_2_SiO_4_:Mn^2+^ (*λ*_em_ = 525 nm/FWHM = 1530 cm^−1^),^[^ ^35^, ^36^ ^]^ BaZnAl_10_O_17_:Mn^2+^ (*λ*_em_ = 516 nm/FWHM = 1170 cm^−1^),^[^ ^37^ ^]^ Zn_4_B_6_O_13_:Mn^2+^ (*λ*_em_ = 540 nm/FWHM = 1100 cm^−1^).^[^ ^38^ ^]^ In corresponding cubic or octahedral ligand fields, the Mn^2+^‐related emission shifts toward the red spectral range as the lowest excited ^4^T_1(_ *_g_*_)_(^4^G) state becomes stabilized due to occupation of the t_2_ *_g_*‐type 3d orbitals by an additional electron. The stronger ligand field and necessary vibronic coupling to break the parity selection rule also results in higher FWHMs for octahedrally or cubically coordinated Mn^2+^ than for the case of tetrahedral coordination. Representative examples for luminescent compounds with octahedrally coordinated Mn^2+^ are MgSiO_3_:Mn^2+^ (*λ*_em_ = 661 nm/FWHM ≈ 2100 cm^−1^),^[^ ^39^ ^]^ Mg_4_Ta_2_O_9_:Mn^2+^ (*λ*_em_ = 670 nm/FWHM ≈ 2000 cm^−1^),^[^ ^36^, ^40^ ^]^ Ba_3_MgSi_2_O_8_:Mn^2+^ (*λ*_em_ = 620 nm/FWHM = 1650 cm^−1^)^[^ ^41^ ^]^ or *α*‐Na_2_CaMg(PO_4_)_2_:Mn^2+^ (*λ*_em_ = 620 nm/FWHM = 2100 cm^−1^).^[^ ^42^ ^]^ Mn^2+^ usually occupies Mg or Zn sites as they match in terms of ionic radius and charge. However, Mn^2+^ is also known to substitute sites occupied by aliovalent ions like Al^3+^,^[^ ^43^, ^44^, ^45^, ^46^, ^47^, ^48^ ^]^ Sc^3+^,^[^ ^49^ ^]^ or Li^+[^ ^44^, ^50^, ^51^, ^52^ ^]^ and may therefore be considered an activator ion in alkali lithooxidosilicates as well. Especially Li sites should be well suited for Mn^2+^ substitution as its ionic radius of 59 pm is de facto identical with those of Mg^2+^ (57 pm) and Zn^2+^ (60 pm) in tetrahedral coordination.^[^ ^53^ ^]^ This idea is also promoted by the known capability of most alkali lithooxidosilicates to compensate a significant amount of charge‐defects, e.g. caused by aliovalent Eu^2+^ activation or Si^4+^ deficit.^[^ ^19^, ^54^ ^]^ Given the comparably small size of Mn^2+^, a higher coordination number than six for this ion is rather uncommon, as also suggested by Pauling's radius ratio rule. Such an example is CaF_2_:Mn^2+^ (*λ*_em_ = 495 nm/FWHM ≈ 1640 cm^−1^) with a comparably high quenching temperature of 580 K related to the large band gap of the compound.^[^ ^55^, ^56^ ^]^ In this work, we follow up on these ideas and investigate the successful class of UCr_4_C_4_‐type alkali lithooxidosilicates as possible host compounds for Mn^2+^. The structures of these compounds comprise both tetrahedrally coordinated sites for the Li^+^ ions and cuboidally coordinated sites for the larger alkali cations that both could be potentially occupied by Mn^2+^. This is not only interesting from a fundamental perspective. In fact, the presence of two potential activator sites that give rise to both green and red emission could lead to warm white light‐emitting phosphors without the need of mixing two different compounds, which is an additional benefit to a more sustainable approach to phosphors.

A series of seven Mn^2+^‐activated alkali lithooxidosilicates was prepared to gain a better understanding of their luminescence properties and to reveal possible trends. We therefore considered exclusively those known compounds featuring cation ordering and excluded those with complex disorders (e.g., K_1.6_Na_2.1_Li_0.3_[Li_3_SiO_4_]_4_ or CsKNaLi[Li_3_SiO_4_]_4_) as a defined coordination environment is crucial for our analysis.^[^ ^24^, ^57^ ^]^

## Results and Discussion

### Structural Features

**Figure 1:**
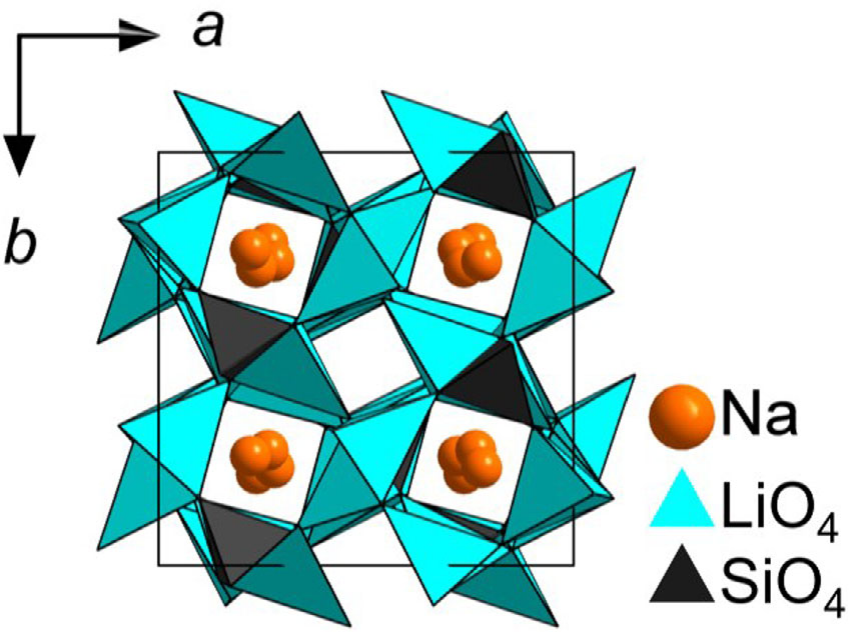
Structural depiction of Na[Li_3_SiO_4_] (view along crystallographic *c* direction) as a representative example of a UCr_4_C_4_‐type compound.

The alkali lithooxidosilicates are structurally derived from the UCr_4_C_4_ structure type (*I*4/*m*, no. 87).^[^ ^58^, ^59^ ^]^ Their structure is composed of corner and edge sharing [LiO_4_]‐ and [SiO_4_]‐tetrahedra, which are arranged in *vierer*‐*ring* channels. The larger alkali metal ions (Na^+^‐Cs^+^) are situated within these channels, occupying sites with distorted cuboidal symmetry. In some cases, Li^+^ is not only part of the tetrahedral backbone but also incorporated within these channels. However, due to the small size of these cations, cubic coordination is avoided and the Li^+^ ions are displaced in an off‐centered fashion to allow square‐planar, fourfold coordination by O^2−^ ligands.^[^ ^22^, ^57^, ^60^, ^61^ ^]^ There are only two known compounds in which all channels are filled with exclusively one sort of alkaline metal ion: Na[Li_3_SiO_4_] (*I*4_1_/*a*, no. 88) and K[Li_3_SiO_4_] (*P* $\bar{1}$, no. 2).^[^ ^59^, ^62^ ^]^ Noticeably, the exchange of Na^+^ for the bigger K^+^ leads to a significant symmetry lowering, which can be related to lattice distortions. Any other alkali lithooxidosilicates contain two different channel types, with one primarily accommodating smaller cations (Li^+^, Na^+^) and the other hosting larger cations (K^+^, Rb^+^, Cs^+^). Complex disordering for the Li^+^ ions is observed if these ions share a channel with larger cations as Li^+^ prefers the sites with approximately square‐planar coordination, whereas the heavier alkaline metal ions occupy the distorted cubic sites.^[^ ^57^, ^61^ ^]^ However, a full occupation of both sites is not feasible due to a to short hypothetical Li^+^ ↔ A^+^ distance (where A = Na, K, Rb, Cs). A representative depiction of the UCr_4_C_4_‐typed alkali lithooxidosilicate Na[Li_3_SiO_4_] is given in Figure 1.

Regarding the incorporation of possible activator ions, it is well‐known that Eu^2+^ occupies exclusively the cuboidally coordinated positions due to its relatively large ionic radius. Mn^2+^ on the other hand is much smaller and may therefore also occupy the tetrahedral Li^+^ positions. Table 1 summarizes the composition and space groups of the phosphors investigated in this study.

**Table 1:** Composition and space groups of the investigated Mn^2+^‐activated alkali lithooxidosilicates.

Compound	Space group	Reference
RbNa_3_[Li_3_SiO_4_]_4_: 0.25% Mn^2+^	*I*4/*m *(no. 87)	[57]
Na[Li_3_SiO_4_]: 0.1% Mn^2+^	*I*4_1_/*a *(no. 88)	[62]
NaK_7_[Li_3_SiO_4_]_8_: 0.1% Mn^2+^	*I*4_1_/*a* (no. 88)	[19, 63]
CsKNa_2_[Li_3_SiO_4_]_4_: 0.2% Mn^2+^	*I*4/*m *(no. 87)	[57]
RbKLi_2_[Li_3_SiO_4_]_4_: 0.1% Mn^2+^	*I*4/*m *(no. 87)	[22]
K[Li_3_SiO_4_]: 0.1% Mn^2+^	*P* $\bar{1}$ (no. 2)	[59]
RbNa[Li_3_SiO_4_]_2_: 0.17% Mn^2+^	*C*2/*m* (no. 12)	[57]

### Luminescence Spectroscopy

**Figure 2:**
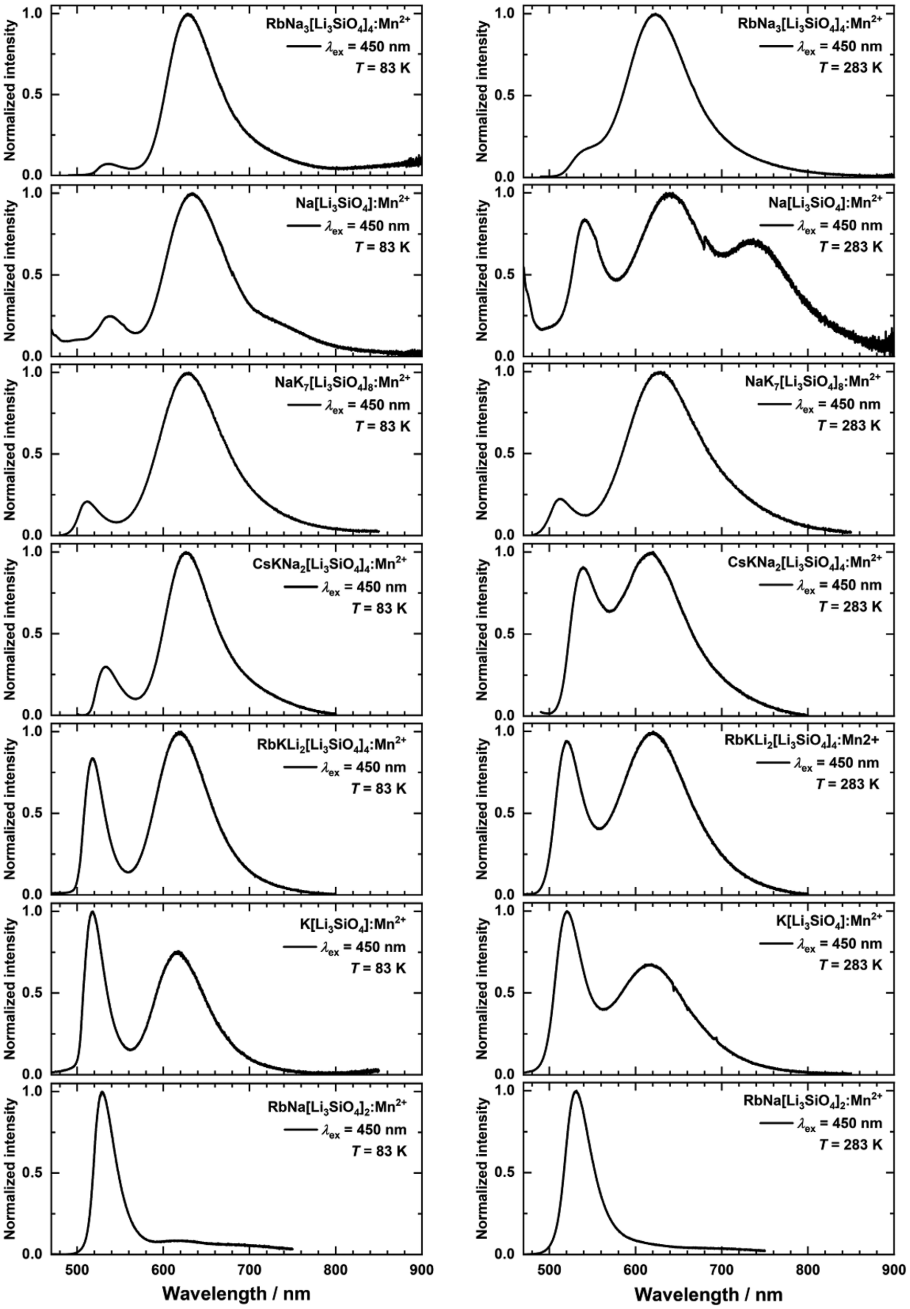
Photoluminescence emission spectra of Mn^2+^‐activated alkali lithooxidosilicates at 83 K (left) and 283 K (right).

All investigated Mn^2+^‐activated alkali lithooxidosilicates (Mn^2+^ content 0.1–0.25 mol%) presented in this work show two distinguishable emission bands located at 515–530 and 610–635 nm, respectively. This indicates that Mn^2+^ occupies two crystallographically independent sites with different coordination number (CN) based on the dependence of the energy of the ^4^T_1(_ *_g_*_)_(^4^G) → ^6^A_1(_ *_g_*_)_(^6^S) transition of Mn^2+^ on the ligand field strength. In general, green emission is observed from Mn^2+^ in tetrahedral O^2−^‐based coordination, whereas coordination numbers of six or eight lead to a red emission. We therefore assign the green emission to Mn^2+^ occupying the tetrahedrally coordinated Li sites and the red emission to Mn^2+^ occupying the cuboidally coordinated alkali sites. This is also in agreement with the width of the emission bands as the comparably large cuboidal sites lead to some flexibility in the Mn─O bonds. The tetrahedrally coordinated Li sites, on the other hand, are particularly small. Any change in the Mn─O distance is thus sterically already limited generally resulting in a comparably narrow emission band. In addition, tetrahedral coordination of Mn^2+^ generally leads to barely any dependence of the energy of the ^4^T_1_(^4^G) state on the ligand field strength according to the Tanabe–Sugano diagram for a 3d^5^ HS ion (Figure S8). This is also indicated by the comparably narrow lowest energetic excitation bands, generally observed for tetrahedrally coordinated Mn^2+^.^[^ ^64^, ^65^ ^]^ However, the intensity ratio of these two bands at both 77 K and room temperature differs drastically, showcasing different site occupation preferences of Mn^2+^ within the investigated compounds. If random occupation of both Li and Na sites is assumed based on the lacking ligand field stabilization energy for a d^5^ HS ion, a more dominant green emission is to be expected as there are more Li sites than Na sites in any of the regarded UCr_4_C_4_‐type alkali lithooxidosilicates. Nevertheless, most considered Mn^2+^‐activated alkali lithooxidosilicates in this work show intense red emission. This may indicate a general preference of Mn^2+^ to occupy the slightly larger Na sites. Another possible explanation for this observation is non‐radiative ${\mathrm{Mn}}_{\left(\mathrm{tetrah}.\right)}^{2+}$→ ${\mathrm{Mn}}_{\left(\mathrm{cubic}\right)}^{2+}$ energy transfer. This, however, seems less likely since the critical distance of the energy transfer for acceptors with strongly forbidden transitions is generally low.^[^ ^66^ ^]^ Moreover, the restriction by both the spin and Laporte selection rule for the ^4^T_1(_ *_g_*_)_(^4^G) ← ^6^A_1(_ *_g_*_)_(^6^S) transition of the cuboidally coordinated Mn^2+^ ions implies a Dexter‐type energy transfer with an exponential distance dependence.^[^ ^67^ ^]^ A possible ${\mathrm{Mn}}_{\left(\mathrm{tetrah}.\right)}^{2+}$→ ${\mathrm{Mn}}_{\left(\mathrm{cubic}\right)}^{2+}$ energy transfer could be identified by concentration‐dependent measurements within future experiments and was not in the scope of this work. Figure 2 compares the emissions of the investigated samples upon 450 nm excitation at 83 and 283 K. The spectra clearly reveal noticeable differences in the intensity ratio between the green and red emission dependent on the composition of the alkali lithooxidosilicate hosts.

Generally, most samples show a predominantly red emission, which is rather surprising if the size mismatch between Na^+^ ($r_{Na^{+}}$ = 1.18 Å) and Mn^2+^ ($r_{Mn^{2+}}$ = 0.96 Å) according to the Shannon radii is considered.^[^ ^53^ ^]^ This might be due to electrostatic repulsion by nearby cations, thereby leading to a preferential occupation of bigger voids with larger mutual cation–cation distances by the higher charged Mn^2+^ ions. However, this apparent mismatch motivated us to have a closer look on the mean effective ionic radii (MEFIR) of the available substitution sites within the investigated compounds. These were calculated in an iterative process starting with M─O distances derived from single‐crystal X‐ray diffraction data.^[^ ^68^ ^]^ The results are summarized and compared in Table 2, showcasing some clear correlations of the emission profiles and the MEFIR of the possible activator sites. First, if effectively smaller Na sites are available, the red component of the emission is dominant, e.g., in Na[Li_3_SiO_4_]:Mn^2+^ (MEFIR(Na^+^) = 111.6 pm) and RbNa_3_[Li_3_SiO_4_]_4_:Mn^2+^ (MEFIR(Na^+^) = 115.4 pm). On the other hand, the absence of smaller Na sites enhances green emission, e.g., in K[Li_3_SiO_4_]:Mn^2+^ (MEFIR(K^+^) = 127.6 pm), RbNa[Li_3_SiO_4_]_2_:Mn^2+^ (MEFIR(Na^+^) = 118.5 pm) and RbKLi_2_[Li_3_SiO_4_]_4_:Mn^2+^ (MEFIR(K^+^) = 142.4 pm). In addition, the red emission band is shifted to longer wavelengths for smaller Na sites. This observation is expected given a stronger ligand field acting at smaller Na sites that should result in a stabilization of the excited ^4^T_1(_ *_g_*_)_(^4^G) state. Another possible explanation for a red‐shifted emission of Mn^2+^ could be an increased degree of covalency in the Mn─O bond.^[^ ^69^ ^]^ However, this is not the case for the Mn^2+^‐activated alkali lithooxidosilicates as the location of the ligand field‐independent ^4^A_1(_ *_g_*_)_,^4^E_(_ *_g_*_)_(^4^G) ↔ ^6^A_1(_ *_g_*_)_(^6^S) transition at 427–430 nm barely changes among the investigated compounds (see Figure S11), which usually reacts sensitively to subtle changes in the interelectronic repulsion and thus, the covalency of the metal─ligand bond.^[^ ^70^ ^]^

**Table 2:** Mean effective ionic radii (MEFIR) of the smallest channel sites of the investigated alkali lithooxidosilicates as well as characteristic luminescence properties. Decay times are amplitude‐weighted average values derived from triexponential fit functions. The quenching temperatures *T*_1/2_ were estimated from temperature dependent decay curves.

Compound	MEFIR (pm)	FWHM (cm^−1^) (*T* = 83 K) green/red	*λ*_em_ (83 K) (nm) green/red	*τ*_green_ (ms) (*T* = 83 K) green/red	Stokes shift (cm^−1^) (*T* = 83 K) green/red	*T*_1/2_ (K) green/red
RbNa_3_[Li_3_SiO_4_]_4_: 0.25% Mn^2+^	132.0 (Na1) 115.4 (Na2)	830/1670	536/629	3.5/15.5	2400/3130	–/470
Na[Li_3_SiO_4_]: 0.1% Mn^2+^	111.6 (Na1)	940/2070	538/634	3.2/5.5	1930/3830	380/325
NaK_7_[Li_3_SiO_4_]_8_: 0.1% Mn^2+^	122.0 (Na1)	930/2120	511/629	5.1/4.2	1190/3040	330/375
CsKNa_2_[Li_3_SiO_4_]_4_: 0.2% Mn^2+^	117.0 (Na1) 145.8 (K1)	920/1790	533/626	4.4/16.5	2270/3220	585/500
RbKLi_2_[Li_3_SiO_4_]_4_: 0.1% Mn^2+^	142.4 (K1)	1060/1920	518/620	5.3/4.3	1700/3920	280/355
K[Li_3_SiO_4_]: 0.1% Mn^2+^	127.6 (K4)	1070/1910	518/616	5.7/4.1	1700/3880	320/370
RbNa[Li_3_SiO_4_]_2_: 0.17% Mn^2+^	118.5 (Na1)	890/–	529/610–620	5.0/9.7	2050/–	490/–

**Figure 3:**
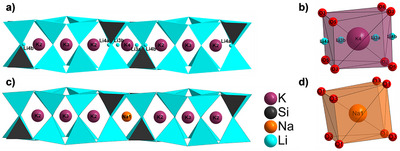
View perpendicular to the *vierer*‐ring channels and depiction of the cations in the cuboidal voids of LiK_7_[Li_3_SiO_4_]_8_ (a+b) and NaK_7_[Li_3_SiO_4_]_8_ (c+d). The Li3, Li4 and K4 sites in a) and b) are only partially occupied.

However, those compounds with exceptionally large activation sites (MEFIR ≥ 122 pm) derive from both trends as they show surprisingly intense red emission. We therefore assume that Mn^2+^ occupies a site that deviates from the cuboidally coordinated Na or K sites. This phenomenon is also well known from Mn^2+^‐activated fluorite‐type compounds, in which the local environment of Mn^2+^ is formally strictly cubic only for CaF_2_ ($r_{Ca^{2+}}$ = 112 pm) and CdF_2_ ($r_{Cd^{2+}}$ = 110 pm).^[^ ^56^ ^]^ Diverging coordination geometries with lower symmetry in SrF_2_ ($r_{Sr^{2+}}$ =  126 pm), PbF_2_ ($r_{Pb^{2+}}$ = 129 pm) and BaF_2_ ($r_{Ba^{2+}}$ = 142 pm) were derived from electron paramagnetic resonance (EPR) data and luminescence spectra, indicating an off‐centered positioning of the Mn^2+^ ion.^[^ ^71^, ^72^, ^73^, ^74^ ^]^ In the alkali lithooxidosilicates, a similar behavior is known from the Li^+^ ions, which is comparable in size to Mn^2+^ ($r_{Li^{+}}$ = 92 pm, $r_{Mn^{2+}}$ = 96 pm).^[^ ^53^ ^]^ Single‐crystal X‐ray diffraction data showed that Li^+^ (if incorporated into the channel) seemingly avoids the cubic sites and occupies sites with square planar symmetry or off‐centered positions without inversion symmetry, respectively.^[^ ^22^, ^57^, ^60^, ^61^ ^]^ A particularly noteworthy example are the otherwise isotypic alkali lithooxidosilicates *M*K_7_[Li_3_SiO_4_]_8_ (*M* = Li, Na), where *M* = Na occupies the center of the distorted cubic position and *M* = Li moves to an off‐centered position (see Figure 3).^[^ ^19^, ^61^ ^]^ Arguably, Mn^2+^ is expected to preferentially occupy the Li sites given the stronger similarity in the ionic radii compared to the difference in ionic radii of eightfold coordinated Mn^2+^ and Na^+^.

**Figure 4:**
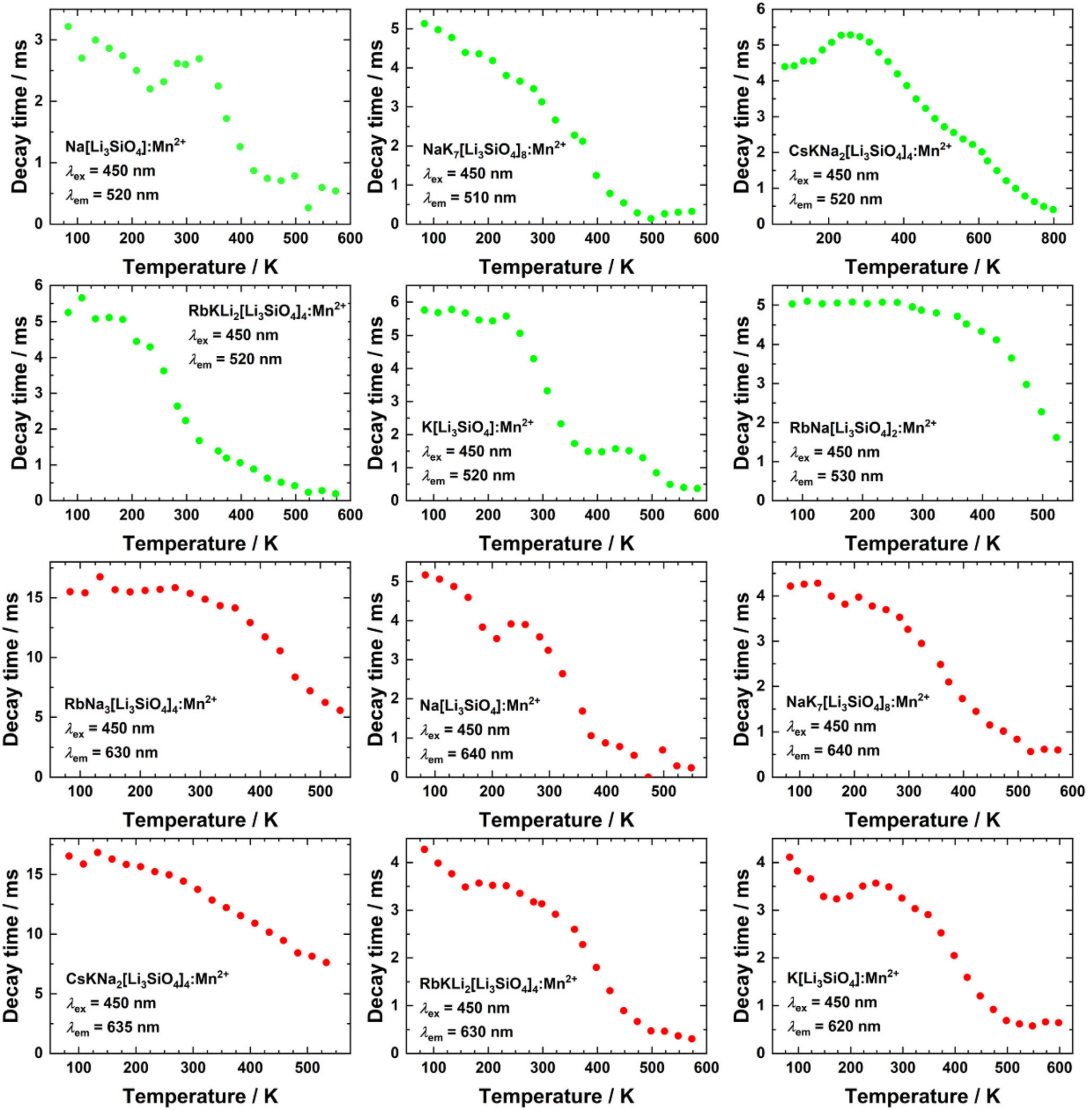
Temperature‐dependent photoluminescence decay times of Mn^2+^‐activated alkali lithooxidosilicates. Decay times are amplitude‐weighted average values derived from triexponential fit functions.

Figure 4 depicts the time‐resolved luminescence data of the regarded Mn^2+^‐activated alkali lithooxidosilicates. The green emission in the range of 520–530 nm for each sample is characterized by decay times in the order of 3–6 ms, which is typical for the spin‐forbidden 3d^5^ (HS) ↔ 3d^5^ (HS) transitions of tetrahedrally coordinated Mn^2+^ due to a relieve in the parity selection rule.^[^ ^75^, ^76^ ^]^ However, the decay times of most red components (Table 1 and Figure 4) are significantly larger (*τ* ∼ 10–20 ms) and thus, rather comparable to values known from Mn^2+^ in a centrosymmetric environment, like octahedrally or cubically coordinated Mn^2+^ (*τ* ∼ 50–200 ms at 77 K).^[^ ^56^, ^77^ ^]^ However, the cuboidally coordinated sites in any of the herein investigated compounds are distorted and thus, the parity restrictions of the 3d^5^ HS ↔ 3d^5^ HS transitions are weakened. In ultimate consequence, the observed decay time is in between values known from tetrahedrally and undistortedly cubically coordinated Mn^2+^. In addition to selection rules affecting the radiative decay, the total decay times might be shortened due to additional nonradiative decay processes such as thermally induced crossover or thermal ionization via the conduction band with subsequent charge carrier trapping. The latter mechanism is also known to correlate with the bandgap of the host compound, due to photoionization as a major quenching mechanism.^[^ ^32^ ^]^ The relatively small bandgap of alkali lithooxidosilicates of ∼ 5.5–6.0 eV may thus also promote a low thermal quenching temperature.^[^ ^21^, ^78^ ^]^ In Na[Li_3_SiO_4_]:Mn^2+^, the cuboidal symmetry at the Na sites is particularly strongly distorted resulting in an unusually short decay time (*τ* = 5.2 ms) at 83 K. The same general trend is observed in alkali lithooxidosilicates with uncommonly large available cuboidally coordinated cation sites in the *vierer*‐ring channels (NaK_7_(Li_3_SiO_4_)_8_, MEFIR = 122.0 pm), RbKLi_2_[Li_3_SiO_4_]_4_, MEFIR = 142.4 pm and K[Li_3_SiO_4_], MEFIR = 127.6 pm). The red luminescence of Mn^2+^ in those compounds is characterized by decay times in the order of ∼ 4–5 ms.

According to temperature‐dependent luminescence measurements (Figure S9), most of the regarded Mn^2+^‐activated alkali lithooxidosilicates show the highest emission intensity in the range of 200 K to 300 K, which is rather unexpected. It may, however, be explained by the participation of trap states that are emptied at a certain temperature and, thus, contribute to the excitation of Mn^2+^, which is also known from defect‐rich halides like Rb_4_CdCl_6_:Mn^2+^.^[^ ^79^ ^]^ Such an interpretation complies with the necessary presence of charge‐compensating vacancies or defects based on the presented aliovalent activation strategy in the alkali lithooxidosilicates. Future experiments will be necessary to elucidate the impact of adjacent vacancies/defects. Another aspect that may contribute to the observed temperature dependencies of both the decay times and the emission intensities is a weakening of the Laporte restriction caused by pronounced vibronic coupling with increasing temperatures. This is particularly prominent in compounds with centrosymmetric lattice sites, like highly symmetric fluoridoperovskites (e.g., KMgF_3_:Mn^2+^) or fluorite (CaF_2_:Mn^2+^).^[^ ^56^, ^77^, ^80^ ^]^ In all regarded Mn^2+^‐activated alkali lithooxidosilicates within this work, thermal quenching already becomes significant in the range of room temperature or slightly above (*T*_1/2_ ∼ 300–500 K), which is unusual compared to many other Mn^2+^‐activated phosphors or Eu^2+^‐activated alkali lithooxidosilicates with quenching temperatures much above 500 K.^[^ ^21^, ^32^, ^33^, ^38^, ^47^ ^]^ This could be an immediate consequence of the comparably low band gaps and the related thermal ionization‐based quenching processes. Two particularly promising candidates within this series are, however, RbNa[Li_3_SiO_4_]_2_:Mn^2+^ and CsKNa_2_[Li_3_SiO_4_]_4_:Mn^2+^, which show thermal quenching temperatures above 450 K for the green and red emission, respectively and, thus, could be promising candidates for future developments of white‐light emitting phosphors.

## Conclusion

The luminescence of seven Mn^2+^‐activated alkali lithooxidosilicates was investigated characterized by a dual‐band emission at both around 530 nm and 620 nm due to the ligand‐field dependent ^4^T_1(g)_(^4^G) → ^6^A_1(g)_(^6^S) transitions of Mn^2+^ occupying both tetrahedrally and cuboidally coordinated alkali sites. The intensity ratio of the two emission bands is shown to be controlled by the composition of the alkali lithooxidosilicate host. There is a general trend that Mn^2+^ has a tendency to occupy the cuboidally coordinated Na sites if they are sufficiently small. The absence of small Na sites, on the other hand, results in the preferred occupation of tetrahedrally coordinated Li sites and a consequent observation of narrow green emission. The unique feature of dual‐band green and red emission makes Mn^2+^ activated alkali lithooxidosilicates intriguing candidates for sustainable one‐component phosphor‐converted white light‐emitting diodes. Notably, many of the herein investigated compounds suffer from a distinct luminescence temperature quenching below 150 °C, which is technologically relevant for that type of application. In that regard, the dual‐band emitting CsKNa_2_[Li_3_SiO_4_]_4_:Mn^2+^ and the narrow‐band green‐emitting RbNa[Li_3_SiO_4_]_2_:Mn^2+^ only show minor thermal quenching relevant for pc‐wLEDs and displays, respectively.

## Conflict of Interests

The authors declare no conflict of interest.

## Supporting information



Supporting Information

## Data Availability

The data that support the findings of this study are available from the corresponding author upon reasonable request.
