# Supplementary material for: Mn2+‐Activated Alkali Lithooxidosilicate Phosphors as Sustainable Alternative White‐Light Emitters
Source: Angew Chem Int Ed Engl. 2025 May 2;64(25):e202504078. doi: 10.1002/anie.202504078 (PMC12171388; doi:10.1002/anie.202504078)
Supplement: Supplementary file 1 — Supporting Information [file ANIE-64-e202504078-s001.docx]

Supporting Information

Mn^2+^-Activated Alkali Lithooxidosilicate Phosphors as Sustainable Alternative White-Light Emitters

**Experimental Section**

**Table S1**. Chemicals used in the preparation of alkali lithooxidosilicates.

| Compound | Quality | Manufacturer | Comment |
| --- | --- | --- | --- |
| Li_2_CO_3_ | 99.999 % | Thermo Scientific |  |
| Na_2_CO_3_ | 99.5 % | Grüssing | Dehydrated at 400 °C |
| K_2_CO_3_ | 99.8 % | J. T. Baker Chemicals | Dehydrated at 400 °C |
| Rb_2_CO_3_ | 99 % | Alfa Aesar | Dehydrated at 400 °C |
| Cs_2_CO_3_ | 99.9 % | Aldrich |  |
| SiO_2_ | 99.99 % | chemPUR | D50<1μm |
| Ethanol | p.a. | Supelco |  |
| N_2_:H_2_ (90:10) | 3.0 | Messer |  |

MnC_2_O_4_ ∙ 2H_2_O:

2.2124 g (15.6 mmol) (NH_4_)_2_C_2_O_4_ ∙ H_2_O were dissolved in 100 ml deionized water and a solution of 2.5383 g (15.0 mmol) MnSO_4._ ∙ H_2_O in 50 ml deionized water was added. A pinkish precipitate forms within 15 minutes. The batch was then stirred and boiled for 15 minutes after which a colorless precipitate remained. The solid was separated by filtration, washed several times with water and dried in a drying cabinet for 12 hours. MnC_2_O_4_ ∙ 2H_2_O was received as colorless powder.^[1]^

General procedures

Any of the investigated Mn^2+^-activated alkali lithooxidosilicates were synthesized by solid state reactions of the corresponding alkali carbonates, SiO_2_ and MnC_2_O_4_ ∙ 2H_2_O. Li_2_CO_3_ was used deficient to open vacancies for occupation by Mn^2+^. The educts were ground in an agate mortar together with some ethanol as dispersing aide. The samples were then dried in a drying cabinet at 80 °C and ground once more. Two or three sintering steps were applied with intermediate grinding. This was found to be necessary as the starting mixtures have a strong tendency to melt if heated immediately over ~ 600 °C. The heating and cooling rates were fixed at 100 °C/h.

Notes on the synthetic procedures

It was found beneficial for phase purity to use slightly excessive amounts of the heavier alkali carbonates (esp. Rb_2_CO_3_). This might be due to the incorporation of small amounts of water or volatilization of the alkali oxides.^[2]^ A reasonable suspicion for volatilization is that prolonged annealing of RbNa[Li_3_SiO_4_]_2_ leads to the formation of RbNa_3_[Li_3_SiO_4_]_4_ as side phase whereas even longer annealing of RbNa_3_[Li_3_SiO_4_]_4_ finally leads to the formation of Na[Li_3_SiO_4_] as side phase.

**Table S2.** Educt stoichiometries and temperature programs used for the synthesis of the investigated Mn^2+^ activated alkali lithooxidosilicates. Heating and cooling rates were fixed at 100 °C/h.

|  | Na[Li_3_SiO_4_] | NaK_7_[Li_3_SiO_4_]_8_ | RbNa_3_[Li_3_SiO_4_]_4_ | RbNa[Li_3_SiO_4_]_2_ | RbKLi_2_[Li_3_SiO_4_]_4_ | CsKNa_2_[Li_3_SiO_4_]_4_ | K[Li_3_SiO_4_] |
| --- | --- | --- | --- | --- | --- | --- | --- |
| Li_2_CO_3_ / mg (mmol) | 442.4 (5.988) | 442.6 (6.988) | 443.4 (6.0) | 221.7 (2.988) | 516.6 (6.993) | 441.6 (5.976) | 442.4 (5.988) |
| Na_2_CO_3_ / mg (mmol) | 221.1 (2.0) | 27.7 (0.26) | 154.2 (1.46) | 51.9 (0.5) |  | 106.0 (1.0) |  |
| K_2_CO_3_ / mg (mmol) |  | 266.2 (1.93) |  |  | 73.6 (0.527) | 69.1 (0.5) | 331.5 (2.4) |
| Rb_2_CO_3_ / mg (mmol) |  |  | 138.6 (0.6) | 138.6 (0.6) | 130.6 (0.57) |  |  |
| Cs_2_CO_3_ / mg (mmol) |  |  |  |  |  | 171.1 (0.525) |  |
| SiO_2_ / mg (mmol) | 240.0 (4.0) | 240.3 (4.0) | 240.3 (4.0) | 120.2 (2.0) | 240.3 (4.0) | 240.3 (4.0) | 240.2 (4.0) |
| MnC_2_O_4_∙2H_2_O / mg (mmol) | 2.2 (0.012) | 2.1 (0.012) | 5.4 (0.03) | 1.8 (0.010) | 2.5 (0.014) | 4.3 (0.024) | 2.2 (0.012) |
| 1. Sintering (air) / °C | 550 | 500 | 650 | 550 | 550 | 700 | 550 |
| Dwell / h | 20 | 2 | 5 | 5 | 20 | 5 | 20 |
| 2. Sintering (air) / °C |  | 650 |  |  |  |  |  |
| Dwell / h |  | 3 |  |  |  |  |  |
| 1. Sintering (N_2_:H_2_) / °C | 750 | 725 | 725 | 725 | 750 | 750 | 750 |
| Dwell / h | 10 | 1 | 1 | 1 | 10 | 1 | 10 |

**X-ray powder diffraction (XRPD) of Mn^2+^-activated alkali lithooxidosilicates**

The purity of the products was determined by XRPD using a Malvern Panalytical X’Pert Pro diffractometer in Bragg-Brentano / reflection geometry (Cu K*_α_* radiation, *λ* = 1.5406 Å). Samples were prepared on an aluminum disc. Rietveld refinement was performed with the program Topas 7.0.0.7 (Bruker ASX, 2022, Germany). Single-crystal diffraction data of K[Li_3_GeO_4_] were used as starting model for the refinement of K[Li_3_SiO_4_]:Mn^2+^. The powder diffraction patterns of the other compounds were refined based on the single-crystal structural data of the respective compounds. Background corrections were performed with Chebychev polynomials of the 20^th^ order. Peak shapes were fitted using the LVol_FWHM_CS_G_L and e0_from_Strain commands, that comprises both Voight and Gaussian components. The diffraction patters are displayed in Figure S1-7. Common side phases are Li_4_SiO_4_ and other alkali lithooxidosilicates. The refinement parameters are given in Table S 3.

**Table S3.** Parameters of the Rietveld refinements of the investigated alkali lithooxidosilicates and database code of the structures used for the refinements.

| Compound | *R*_Bragg_ / % | *R*_exp_ / % | *R*_wp_ / % | *R*_p_ / % | *G.o.o.F.* | Database code |
| --- | --- | --- | --- | --- | --- | --- |
| RbNa_3_[Li_3_SiO_4_]_4_: 0.25 % Mn^2+^ | 3.45 | 3.80 | 10.52 | 7.64 | 2.77 | CCDC 1635539 |
| Na[Li_3_SiO_4_]: 0.1 % Mn^2+^ | 3.35 | 4.16 | 11.03 | 7.84 | 2.66 | COD 1518159 |
| NaK_7_[Li_3_SiO_4_]_8_: 0.1 % Mn^2+^ | 6.19 | 5.46 | 10.58 | 8.24 | 1.94 | CCDC 1861384 |
| CsKNa_2_[Li_3_SiO_4_]_4_: 0.2 % Mn^2+^ | 6.73 | 5.28 | 13.89 | 9.77 | 2.63 | CCDC 1635538 |
| RbKLi_2_[Li_3_SiO_4_]_4_: 0.1 % Mn^2+^ | 6.10 | 4.16 | 12.04 | 8.27 | 2.90 | CCDC 1936593 |
| K[Li_3_SiO_4_]: 0.1 % Mn^2+^ | 8.76 | 5.24 | 16.49 | 11.18 | 3.15 | COD 1536511 |
| RbNa[Li_3_SiO_4_]_2_: 0.17 % Mn^2+^ | 7.09 | 5.35 | 14.67 | 10.92 | 2.74 | CCDC 1635540 |


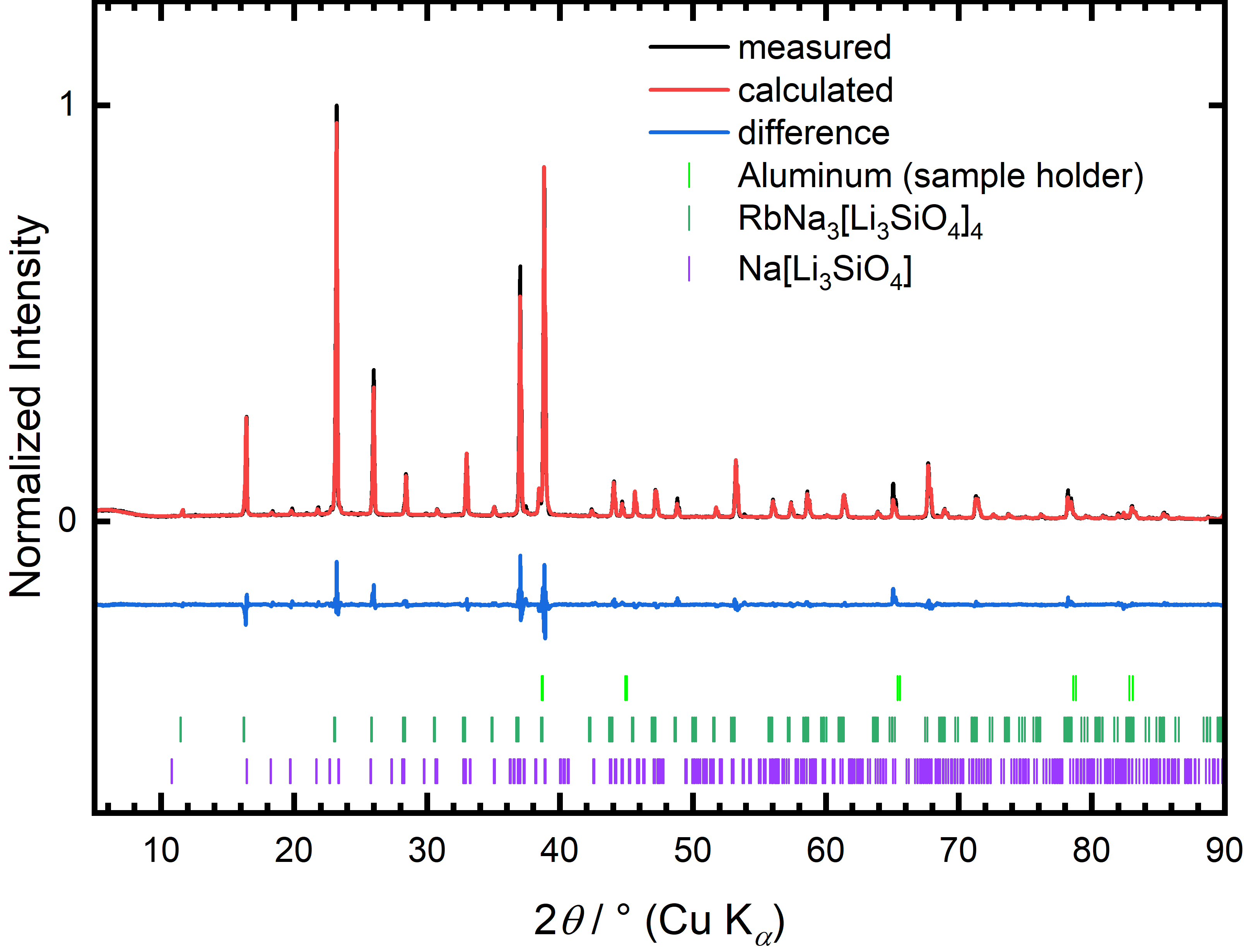


**Figure S1.** Rietveld refined X-ray powder diffraction (XRPD) pattern of RbNa_3_[Li_3_SiO_4_]_4_: 0.25% Mn^2+^.


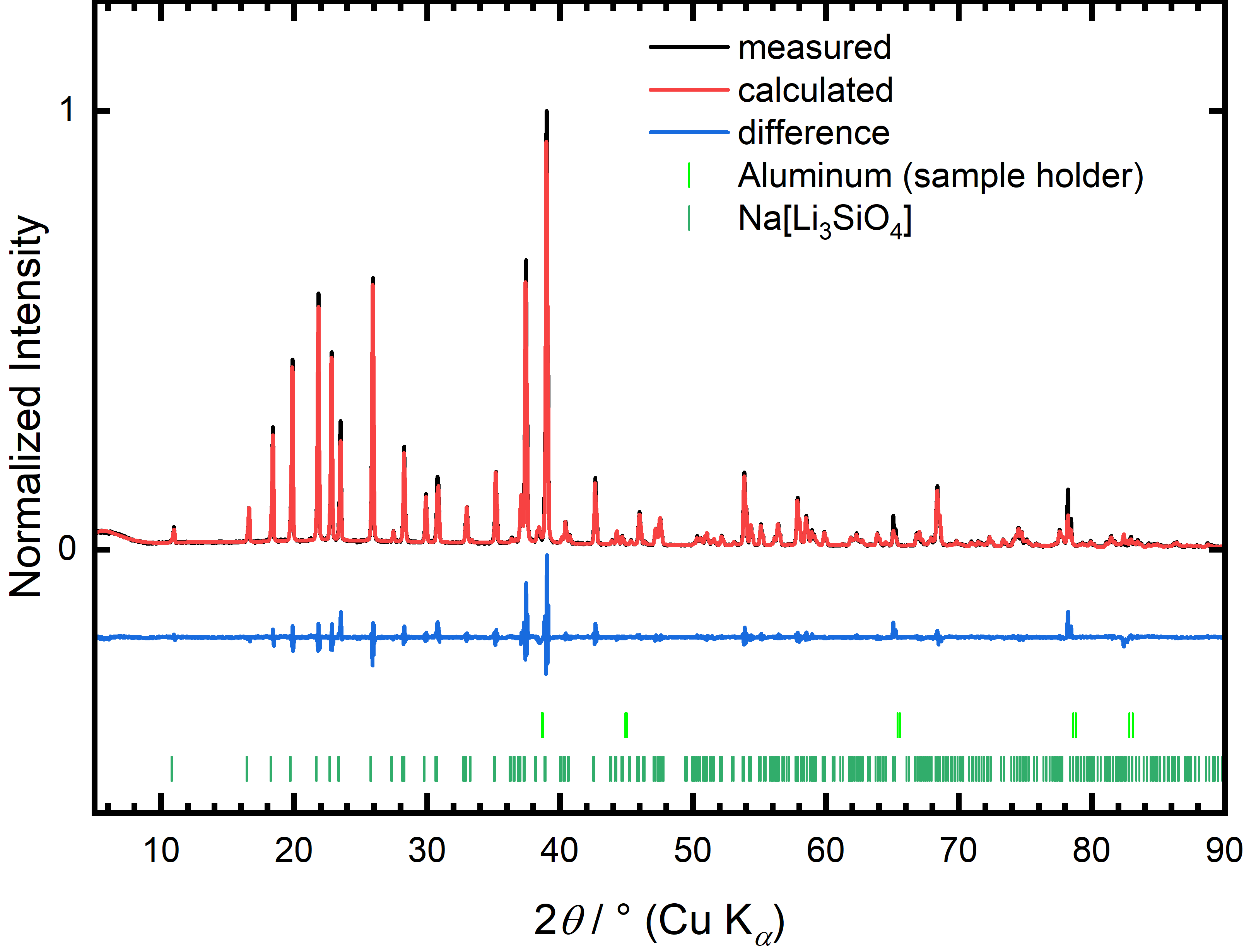


**Figure S2.** Rietveld refined X-ray powder diffraction (XRPD) pattern of Na[Li_3_SiO_4_]: 0.1% Mn^2+^.


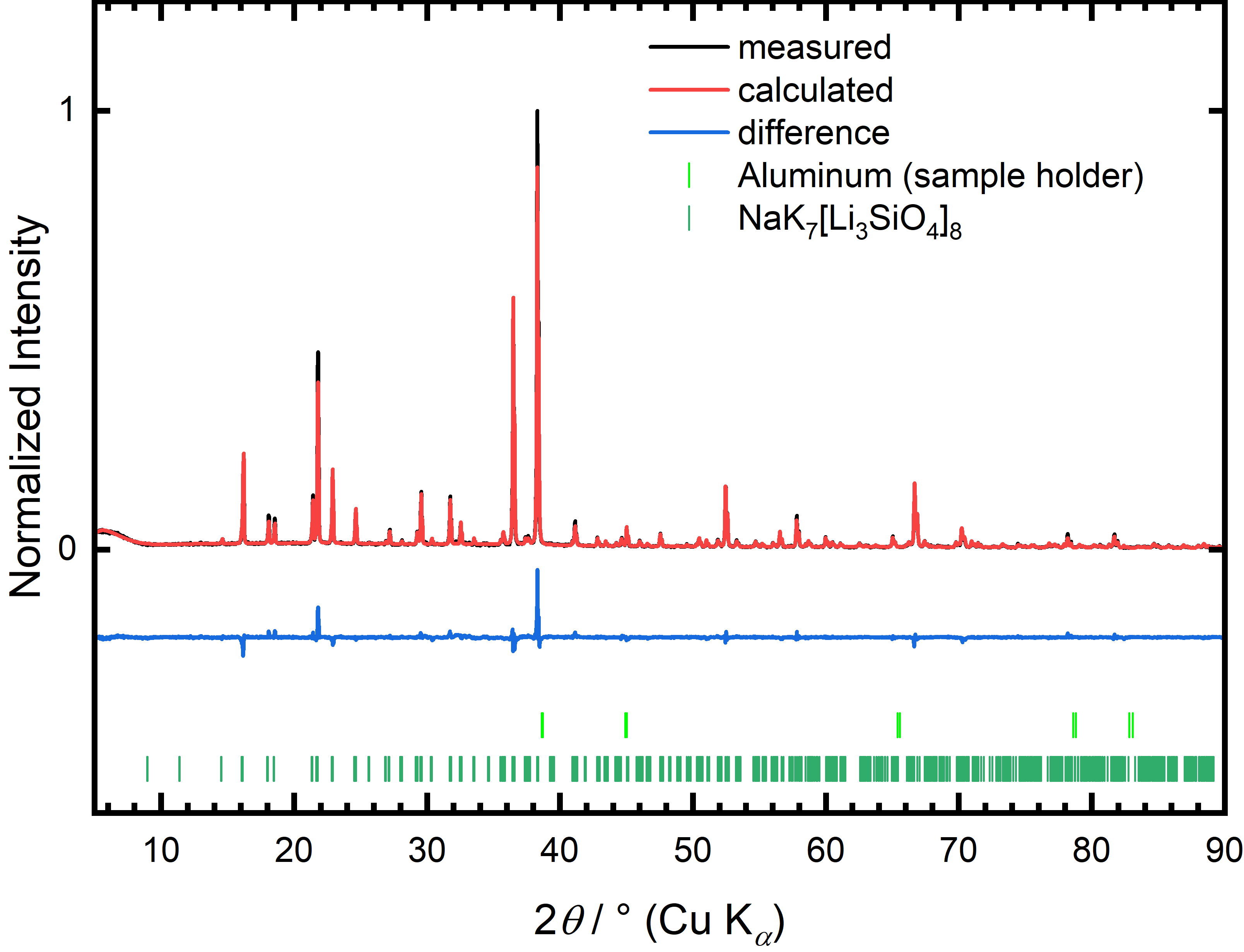


**Figure S3.** Rietveld refined X-ray powder diffraction (XRPD) pattern of NaK_7_[Li_3_SiO_4_]_8_: 0.1% Mn^2+^.


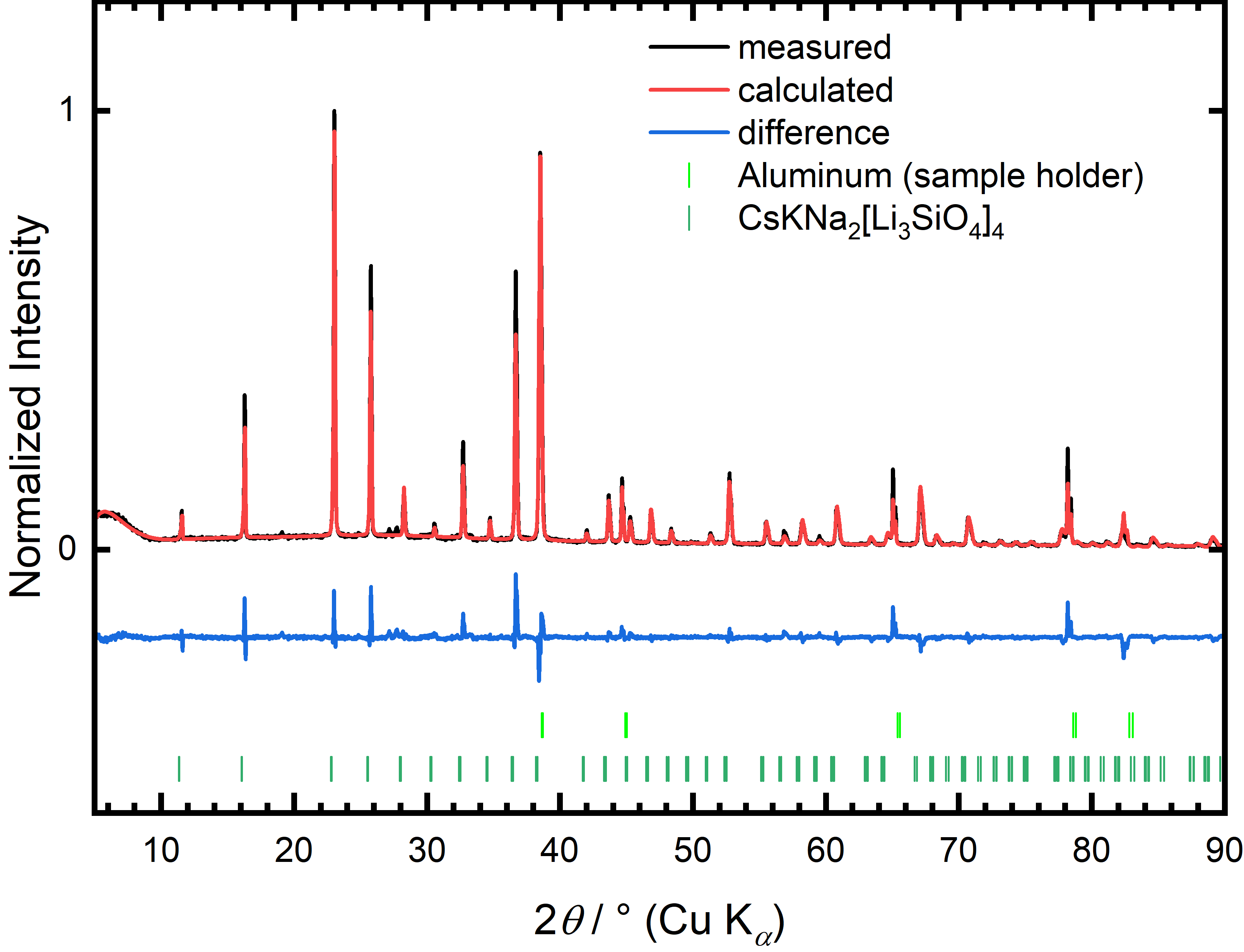


**Figure S4.** Rietveld refined X-ray powder diffraction (XRPD) pattern of CsKNa_2_[Li_3_SiO_4_]_4_: 0.1% Mn^2+^.


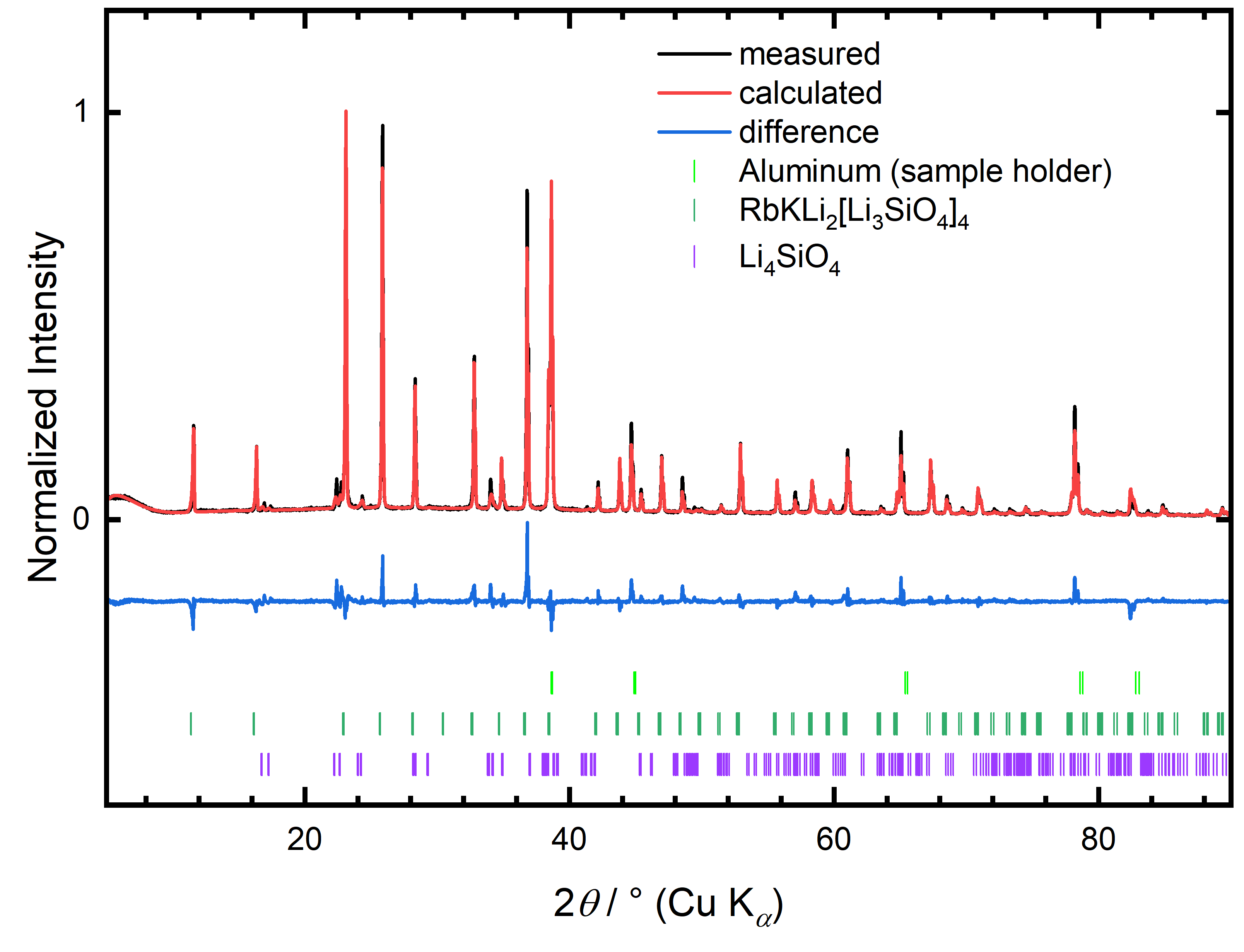


**Figure S5.** Rietveld refined X-ray powder diffraction (XRPD) pattern of RbKLi_2_[Li_3_SiO_4_]_4_: 0.1% Mn^2+^.


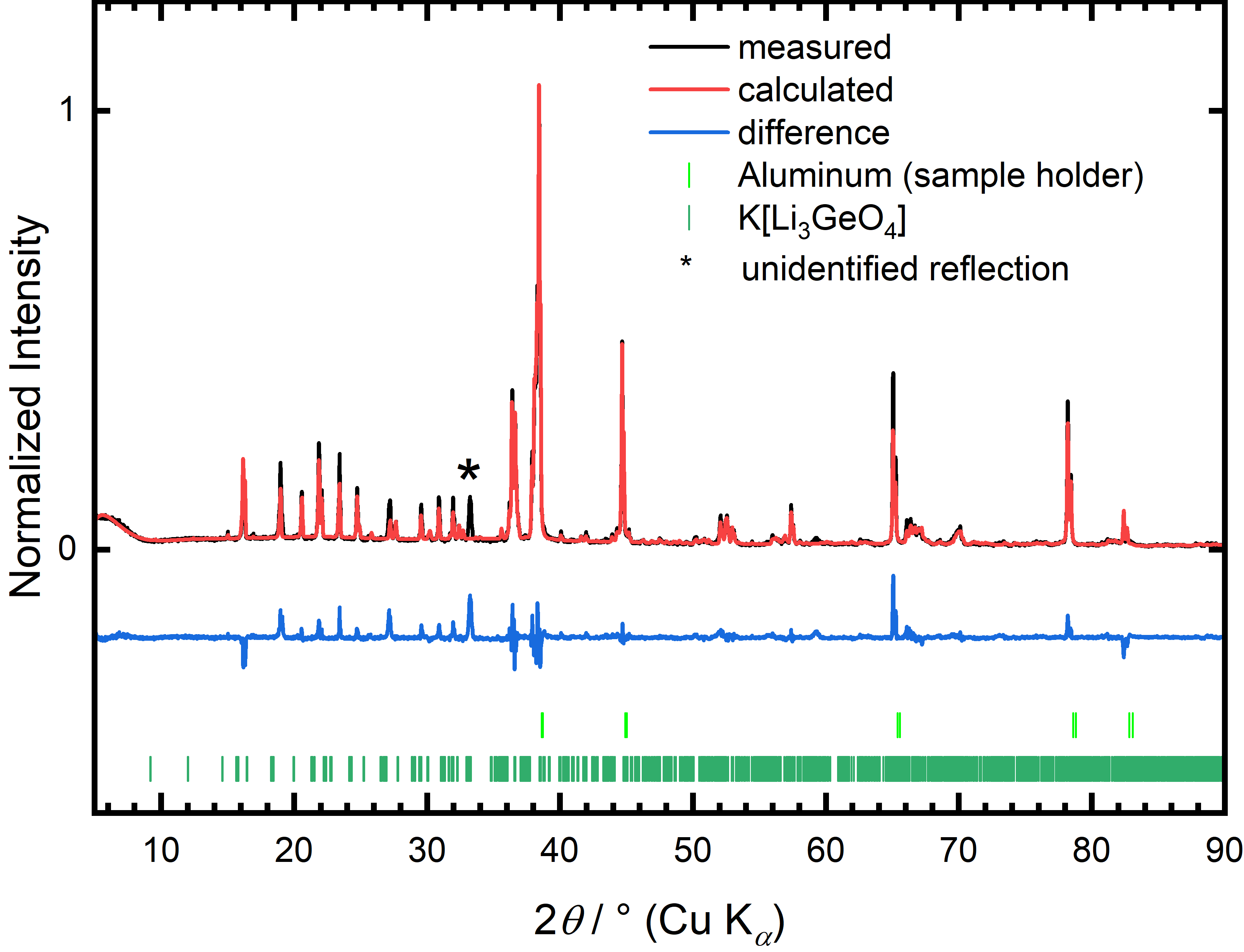


**Figure S6.** Rietveld refined X-ray powder diffraction (XRPD) pattern of K[Li_3_SiO_4_]: 0.1% Mn^2+^.


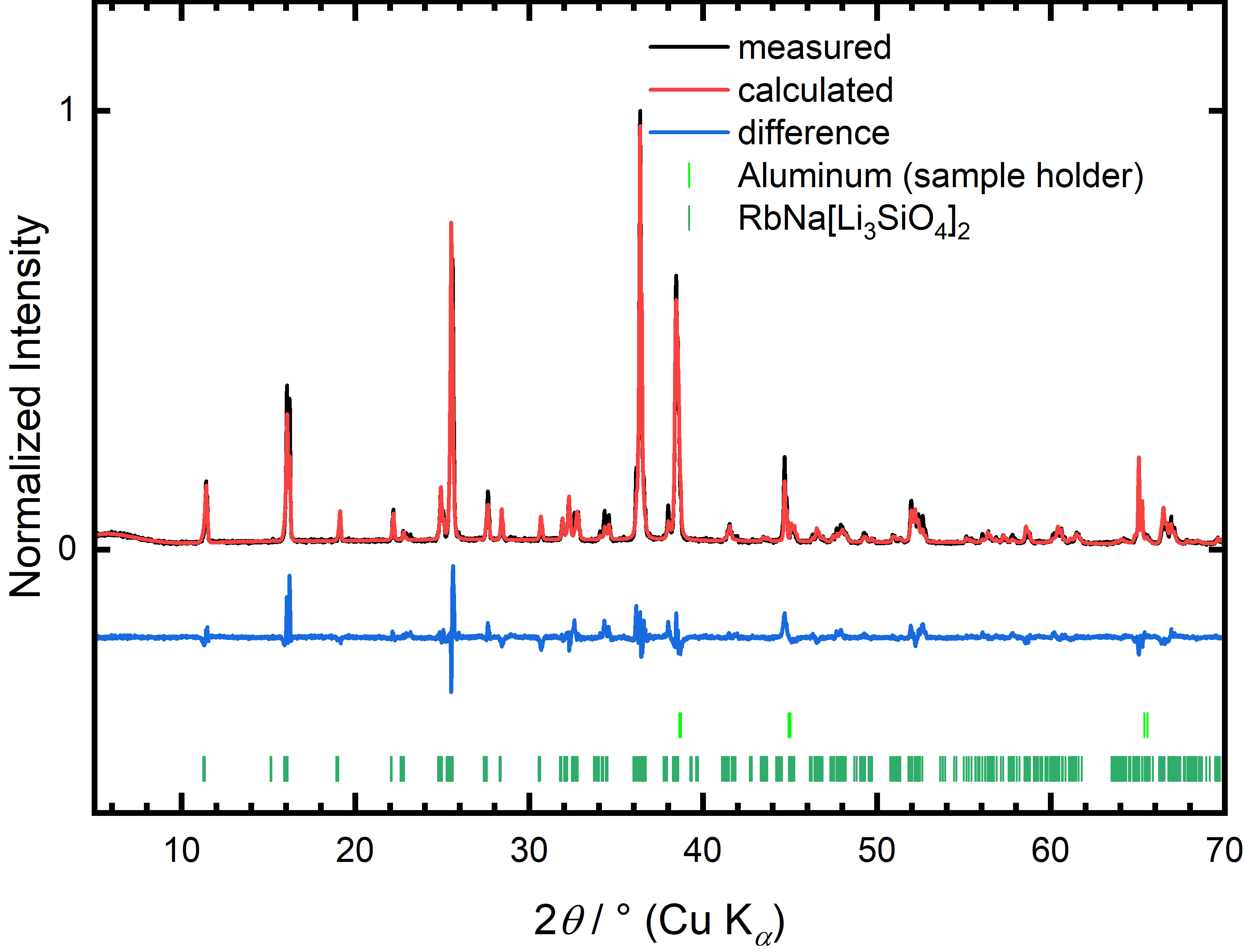


**Figure S7.** Rietveld refined X-ray powder diffraction (XRPD) pattern of RbNa[Li_3_SiO_4_]_2_: 0.17% Mn^2+^.

**Luminescence Spectroscopy**

All samples were sealed in evacuated thin, optically transparent quartz ampoules (ilmasil® quality) to avoid extensive contact of the samples to air and humidity. Optical measurements were performed on an Edinburgh Instruments FLS1000 photoluminescence spectrometer equipped with a 450 W Xe arc lamp as a wavelength-variable excitation source for excitation spectra, double excitation and emission monochromators in Czerny-Turner configuration and a thermoelectrically cooled (−20 °C) photomultiplier tube PMT-980 (Hamamatsu). The emission spectra were corrected with respect to the grating efficiency and PMT sensitivity, while excitation spectra were additionally corrected with respect to the lamp intensity. The decay traces were measured with a laser diode VPL-450 (Edinburgh Instruments, 53.0 mW average incident peak power, l = 450.9 nm) with adjustable temporal pulse width (0.1 µs...1 ms) and variable trigger frequency (0.1 Hz...5 MHz) as the pulsed excitation source. The laser was also used in a continuous wave excitation mode for recording emission spectra. The detection mode for time resolved measurements was single-photon multichannel scaling. Temperature-dependent measurements were performed with a Linkam Scientific THMS600 temperature cell with temperature accuracy of ± 0.1 °C in the regarded temperature range between -190 °C and 525 °C. Sample-ampules where mounted in a custom-made solid silver sample holder with some thermal conductive grease. After each heating or cooling step, a five-minute equalization time was set before a measurement was started.


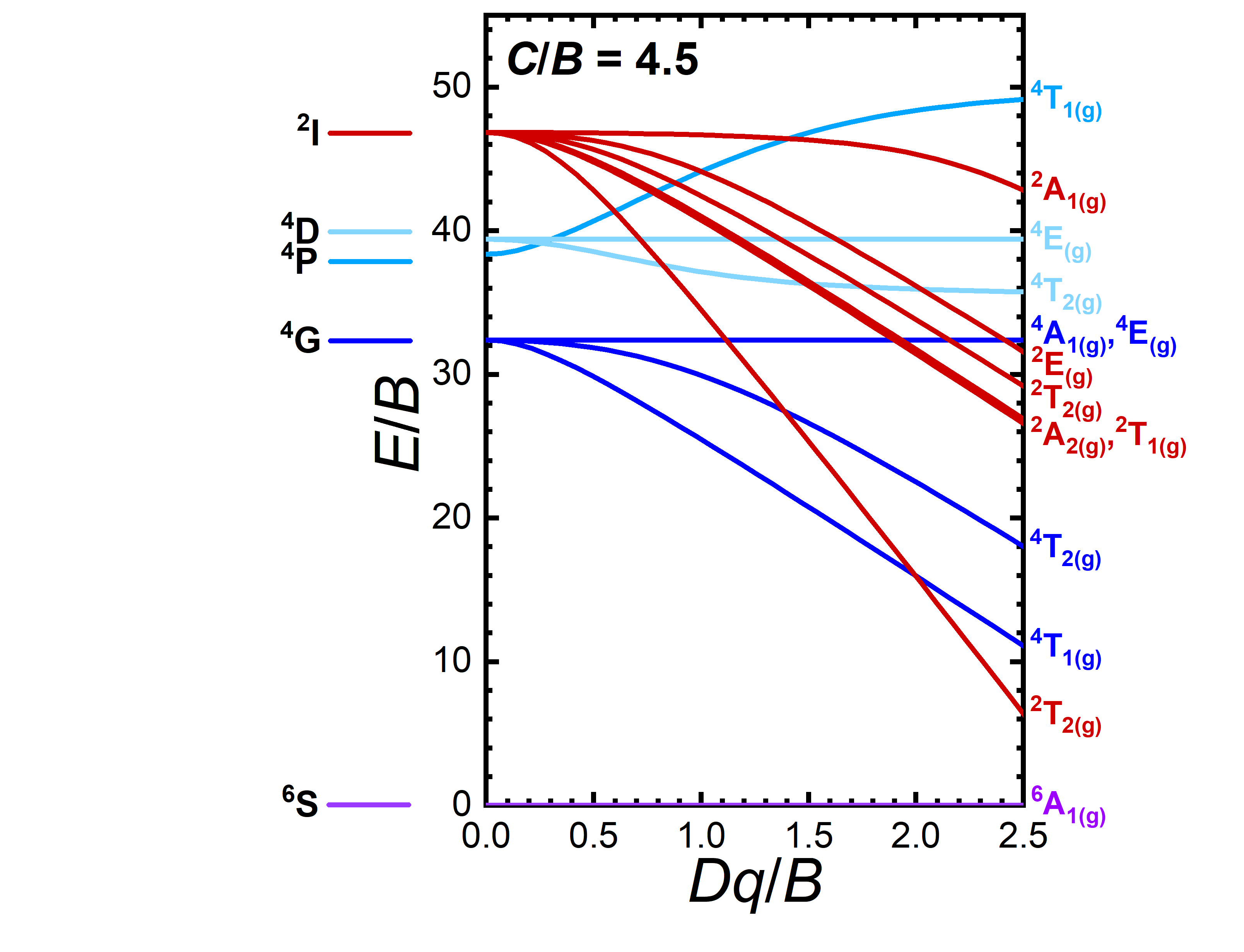


**Figure S8.** Tanabe-Sugano diagram for octahedrally coordinated d^5^ ions. *B* and *C* denote the interelectronic repulsion Racah parameters.





**Figure S9.** Temperature dependence of the emission intensity (full emission range used for integration).





**Figure S10.** Photoluminescence decay curves of Mn^2+^-activated alkali lithooxidosilicates at 83 K. Excitation and emission wavelengths are indicated in the graph.





**Figure S11.** Photoluminescence excitation spectra of Mn^2+^-activated alkali lithooxidosilicates at 83 K. Assignment of states according to the Tanabe-Sugano diagram.

**Structural depiction of RbNa[Li_3_SiO_4_]_2_**


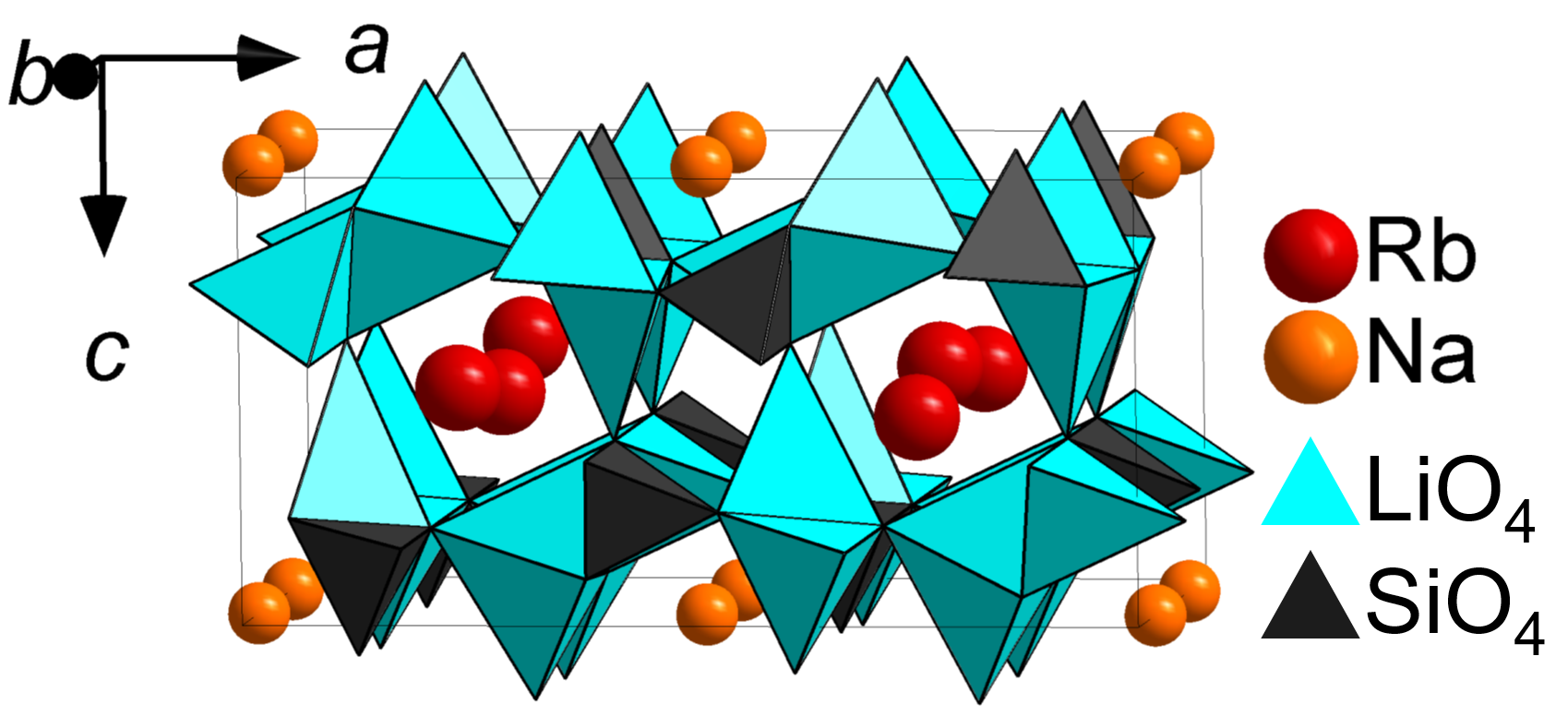


**Figure S12.** Structural depiction of RbNa[Li_3_SiO_4_]_2_ as a representative example of a UCr_4_C_4_-type compound with lower symmetry and different occupation of the channel positions.

[1] A. Huizing, H. A. M. van Hal, W. Kwestroo, C. Langereis, P. C. van Loosdregt, "Hydrates of manganese (II) oxalate" *Mater. Res. Bull.* **1977**, *12*, 605–611.

[2] R. H. Lamoreaux, D. L. Hildenbrand, "High Temperature Vaporization Behavior of Oxides. I. Alkali Metal Binary Oxides" *J. Phys. Chem. Ref. Data* **1984**, *13*, 151–173.
